# Impact of a community contraceptive counselling intervention on adolescent fertility rates: a quasi-experimental study

**DOI:** 10.1186/s12889-019-8122-1

**Published:** 2020-01-08

**Authors:** Elia Diez, Maria J. Lopez, Gloria Perez, Irene Garcia-Subirats, Laia Nebot, Ramon Carreras, Joan R. Villalbi

**Affiliations:** 1 Agència de Salut Pública de Barcelona, Pl Lesseps 1, 08023 Barcelona, Spain; 2 Universitat Autònoma de Barcelona. Campus de la UAB, Pl Cívica s/n., 08193 Bellaterra, Spain; 3 CIBER de Epidemiología y Salud Pública (CIBERESP), Centro de Investigación Biomédica en Red. Instituto de Salud Carlos III, C/ Monforte de Lemos 3-5, 28029 Madrid, Spain; 4 Institut d’Investigació Biomèdica Sant Pau, C/ Sant Antoni Maria Claret 167, 08025 Barcelona, Spain; 5 Universitat Pompeu Fabra, Plaça de la Mercè 10, 08002 Barcelona, Spain; 6 CEPS Salut. C/ Doctor Santponç¸, 60, 08030 Barcelona, Spain; 7 Hospital del Mar, Pg Marítim 25, 08003 Barcelona, Spain

**Keywords:** Health inequalities, Neighbourhood/place, Public health policy, Reproductive health, Adolescence

## Abstract

**Background:**

From 2000 to 2008, in urban areas in Spain, adolescent fertility and abortion rates underwent unprecedented increases, consecutive to intensive immigration from developing countries. To address unmet needs for contraception information and services, a community-based, gender-sensitive and culturally adapted brief counselling intervention (*SIRIAN program*) was launched in some deprived neighbourhoods with a high proportion of immigrants in Barcelona. Once a randomized controlled trial demonstrated its effectiveness in increasing the use of contraceptives, we aim to examine its population impact on adolescent fertility rates.

**Methods:**

Quasi-experimental study with comparison group, using population data from 2005 to 2016. Five neighbourhoods in the lowest tercile of Disposable Household Income were intervened in 2011–13. The comparison group included the three neighbourhoods which were in the same municipal district and in the lowest Disposable Household Income tercile, and displayed the highest adolescent fertility rates. Generalized linear models were fitted to assess absolute adolescent fertility rates and adjusted by immigrant population between pre-intervention (2005–10) and post-intervention periods (2011–16); Difference in Differences and relative pre-post changes analysis were performed.

**Results:**

In 2005–10 the intervention group adolescent fertility rate was 27.90 (per 1000 women 15–19) and 21.84 in the comparison group. In 2011–16 intervention areas experienced great declines (adolescent fertility rate change: − 12.30 (− 12.45 to − 12.21); *p* < 0.001), while comparison neighbourhoods remained unchanged (adolescent fertility rate change: 1.91 (− 2.25 to 6.07); *p* = 0.368). A reduction of − 10.97 points (− 13.91 to − 8.03); *p* < 0.001) is associated to the intervention.

**Conclusion:**

Adolescent fertility rate significantly declined in the intervention group but remained stable in the comparison group. This quasi-experimental study provide evidence that, in a country with universal health coverage, a community counselling intervention that increases access to contraception, knowledge and sexual health care in hard-to-reach segments of the population can contribute to substantially reduce adolescent fertility rates. Reducing adolescent fertility rates could become a feasible goal in cities with similar conditions.

## Background

Pregnancy and childbearing in adolescence increase the risks of social, economic and health problems for mothers, babies and families [1]. Associated with socioeconomic growth [2, 3] world adolescent fertility rates (AFR) have steadily declined in the twenty-first century [4] due to societal changes as the postponement of the completion of education, leaving home, starting employment and settling with a partner, and increased global access to reliable contraception [5–7]. While the worldwide AFR trend is positive, large geographical, socioeconomic, racial and ethnic inequalities persist [1], and increased population mobility adds complexity to this worldwide challenge.

In the first decade of this century, Barcelona and other cities in Spain, a country with universal access to publicly funded health care, including family planning services, underwent an increase in AFR (1999: 3.6 per 1000 women 15–19; 2004: 6.8 per 1000 women 15–19) and adolescent abortion rates (1999: 10.9 per 1000 women 15–19; 2004: 16.2 per 1000 women 15–19) [8]. These data were associated with unprecedented increases in economic immigration from developing countries (city immigrant population 2001: 4.9%; 2010: 17.6%) [9]: even though AFR in the city remained relatively low, a closer look revealed unmet needs for information and services, particularly among immigrant communities [10] (AFR in Barcelona 2005: 8.6 per 1000 women 15–19; in immigrants from developing countries: 29.6 per 1000 women 15–19) (adolescent abortion rates in Barcelona 2005: 15.5 per 1000 women 15–19; in immigrants from developing countries: 31.9) [11]. Newcomers settled in neighbourhoods with low socioeconomic level, which already presented high AFR [12]. Barcelona, with 1.6 million inhabitants, is divided into 73 neighbourhoods of varying size, according to their cultural and geographical characteristics. The neighbourhoods are grouped into 10 municipal districts, which have political representation and administrative responsibilities.

To address this need, Barcelona public health services were commissioned to develop and evaluate a pilot reproductive health preventive intervention. After a review of the main literature [13, 14] the SIRIAN program, a community brief counselling intervention involving public health and health care services, municipal agencies and community partners, was developed and launched in five deprived neighbourhoods where public audiences had identified action on unplanned pregnancies as a priority. A previous randomized clinical controlled trial and pre-post studies have shown its effects in increases in optimal contraceptive use among adult and adolescent participants [15, 16]. The aim of this study is to evaluate the population impact in AFR with a quasi-experimental design, by comparing the changes in intervened and comparison neighbourhoods over 12 years.

## Methods

This is a quasi-experimental study, with a comparison group and pre and post-intervention measures [17]. We used population data from 2005 to 2016 from the Barcelona Municipal Statistics and municipal reports of Disposable Household Income [18], defined as the sum of household final consumption expenditure and savings, minus the change in net equity of households in pension funds [19]. Birth data comes from the Statistical Institute of Catalonia, who elaborates a database of annual births of women based on the civil registry. These databases are provided annually to the Public Health Agency of Barcelona, where they are geocodified to prepare the City Birth Register [20]. Population data for the 73 neighbourhoods comes from the Department of Statistics of the City Council of Barcelona. Fertility data by neighbourhoods must be requested to the Public Health Agency of Barcelona.

The intervention was framed in an urban equity approach strengthening the health and well-being of low income communities and enhancing local community capacity. It was based in evidence [13, 14, 21] theory [22, 23] and a qualitative research [8, 15]. The program was led by community health task groups set in the neighbourhoods, linked to service provision care and supportive environments, and provided by NGO and public health workers.

The five neighbourhoods which received the SIRIAN program were selected after the public identification of unplanned and teenage pregnancy as priorities to be addressed [24, 25]. The comparison group included neighbourhoods meeting the following criteria: 1) they were in the same municipal districts, 2) they were in the same Disposable Household Income tercile, 3) within these, they had the highest AFR and 4) they had enough population to be compared with the intervention group (Table 1). The intervention was carried out from October 2010 to July 2013 in the neighbourhoods of Ciutat Meridiana, Torre Baró and Vallbona, and from September 2011 to October 2014 in El Bon Pastor and Baró de Viver. Common years with intervention in the five neighbourhoods were 2011–13.

**Table 1:** Household Disposable Income, adolescent fertility rate, population count and immigrant percentage by neighbourhood intervention group. Barcelona, 2011

Neighbourhood	Household Disposable Income^a b^	Adolescent fertility rate (number of births)^c^	Neighbourhood population^b^	Neighbourhood immigrant population %^b^
Intervention group				
Vallbona	53.2	19.23 (2)	1331	11.8
Baró de Viver	50.8	16.57 (3)	2366	7.1
Torre Baró	54.5	25.51 (5)	2168	10.1
El Bon Pastor	63.0	23.09 (16)	12,139	14.4
Ciutat Meridiana	39.9	33.96 (27)	10,890	35.6
Total	52.3	26.92 (53)	28,894	21.4
Comparison group				
Les Roquetes	53.8	27.48 (31)	16,009	21.6
La Trinitat Nova	40.3	23.03 (12)	7707	19.1
El Turó de la Peira	58.6	22.73 (23)	15,160	23.1
Total	50.9	24.80 (66)	38,876	21.7
Barcelona	100	8.53	1,620,292	21.5

^a^ Household Disposable Income: Sum of household final consumption expenditure and savings, minus the change in net equity of households in pension funds (Barcelona = 100). OECD (2016). Household disposable income (indicator). doi: 10.1787/dd50eddd-en

^b^ 2011

^c^ 2008–10

The SIRIAN program provided standardized contraceptive counselling to participants. The individual counselling sessions lasted up to 45 min, depending on need. The interview was based on WHO guidelines and communication tools [21, 26]. Sessions were structured according to social cognitive theory, based in motivational interviewing [14, 22, 23, 27, 28] and followed a guide with key components communicated to all participants. The intervention was culturally adapted in accordance with the results of a broad formative investigation. All materials for users were translated and culturally adapted. Contraceptive methods effectiveness and availability, along with tailored counselling was provided in individual sessions in community facilities such as youth centres, libraries and other settings by trained public health nurses or psychologists [29].

In the logic model underlying the intervention, inputs were: a) a 45 min of evidence based and motivational contraceptive counselling, b) trained professionals, c) a small incentive for each participant, d) community facilities, such as libraries or youth centres, where the counselling was delivered, and e) a local community group leadering the implementation of the intervention in each neighbourhood. The interview was designed and delivered to reduce misconceptions, to increase knowledge on contraceptives, condoms, emergency contraception and abortion, and the facilities where contraception, support and health care services were available in the neighbourhood. The interview included reviewing attitudes and beliefs related with sexuality, reproduction and contraception, and enhancing self-efficacy and skills on condom use. These individual factors, together with making visible the accessibility to sexual and reproductive health resources in the community, as well as an improvement of social norms and support to contraceptive use in the neighbourhoods should result in increases of consistent use of modern contraception. As a result, we expected reductions in unintended pregnancies and adolescents’ births and, at long-term, broader social and health impacts, as well as health equity [15, 16, 30].

In the intervention areas, the participants were recruited through leaflets and posters in the streets, and referrals from civic, community and primary care health centres. Participants were asked to invite neighbours, relatives and friends. Eligible participants were women 14 to 49 years old and men 14 and 39 years old. Those who had undergone an irreversible contraceptive method and those who wanted a pregnancy were excluded. The program reached a 21.6% of the age group population in the intervention neighbourhoods. A 55.1% of the participants were girls. A 44.9% were immigrants, while the immigrant population percentage in the intervention neighbourhoods was 21.4% in 2011. The intervention was more effective among immigrant and male adolescents [15, 16]. The protocol was approved by the institutional ethics committee and was carried out in accordance with the principles of the Declaration of Helsinki. All participants signed an informed consent.

The analysis was done at population level. The outcome variable was the annual AFR for each intervention group. The exposure was the intervention implementation, and the covariates were the annual percentages of total immigrant population in each group. The exposition period was 2011–13, the common years of intervention, although the program was carried out from January 2011 to July 2013 in the neighbourhoods of Ciutat Meridiana, Torre Baró and Vallbona, and from September 2011 to October 2014 in El Bon Pastor and Baró de Viver, in order to compare it with the birth data, only available for complete years. Thus, the intervention period October 2010–December 2010 in Ciutat Meridiana, Torre Baró and Vallbona was not included in the intervention period, and part of the intervention was carried out beyond 2013. The equation model was:

$$ Y={\beta}_0+{\beta}_T\left[ Time\right]+{\beta}_I\left[ Intervention\right]+{\beta}_{T\ast I}\left[ Time\ast Intervention\right]+{\beta}_c\left[ Covariate\right]+\varepsilon, $$

where Y: annual AFR; β_**0**_: baseline average of the model (constant); β_T_ [Time]: pre-intervention period (2005–10) and post-intervention period (2011–16); β_**I**_[Intervention]: Intervention and comparison groups; β_T*I_ [Time*Intervention]: interaction between period and intervention; β_**c**_ [Covariate]: annual percentage of immigrant population; ε: error. DiD was implemented as β_T*I_ [Time*Intervention], the interaction term between time and intervention group dummy variable. β coefficients and their 95% CI represent the units of change in AFR per 1000 women 15–19 years old.

We performed a Differences in Differences analysis (DiD) [31] between the pre-intervention (2005–10) and post-intervention periods (2011–16), because the time frame in which changes were expected was longer than the strict period of implementation. This provided also a greater number of years to compare and more data stability. Fertility data for the current Barcelona neighbourhoods were only available since 2007, because in 2006 the municipality changed the former distribution of 38 neighbourhoods into a new one of 73 new neighbourhoods. As adolescent fertility rates for the new 73 neighbourhoods had been retrospectively recalculated by the Barcelona Reproductive Health Information System for 2005 and 2006, we included these 2 years in this study.

Absolute and adjusted changes were calculated between AFR before (2005–10) and post-intervention (2011–16) with regression models with robust SE. By constructing GEE models, an extension of the generalized linear model used in the analysis of correlated longitudinal data, we studied the changes in AFR for the intervention and comparison groups, while controlling the correlation emanating from the repeated nature of the observations compiled in neighbourhoods. Significance testing was two-tailed and the significance level was set at 5%. The analyses were adjusted by the annual percentage of immigrant population in both groups to control for changes in the population. We performed also Poisson regression analysis to provide relative risks through the exponents of coefficients of the model.

Even though the DID analysis was performed between the periods 2005–10 and 2011–16, we segmented the available data (2005–16) in four periods of 3 years (A: 2005–07; B: 2008–10; C: 2001–13 and D: 2014–16) in order to visually explore the trends of A.

FR before and after the intervention. We analysed differences between the periods A and B to study the parallel trends assumption, and also differences between periods C and D to explore the evolution of the effects through regression models. We assessed the trends for groups and the interactions between the period and the group, with their 95% CI.

We performed a sensitivity check to see if the assumptions of the difference in difference design held. Thus, we replicated the model equation and analysis with a comparison group including the rest of neighbourhoods in the lowest Disposable Household Income tercile (19 neighbourhoods included, excluding the 5 which were in the intervention group from a total of the 23 most deprived neighbourhoods in the city), which provided better data stability and allowed assessing the parallel trend assumption, as well as the effects of the intervention.

## Results

**Fig. 1:**
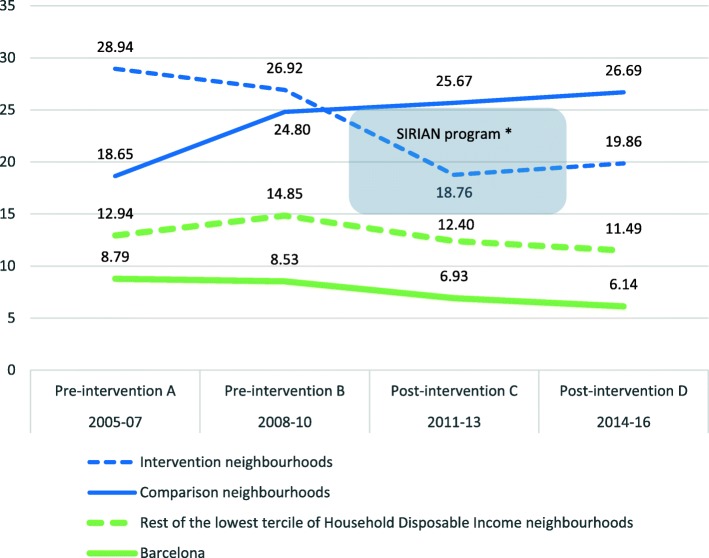
Adolescent fertility rates (per 1000 women 15–19 years) in the SIRIAN program intervention, comparison and rest of low Family Disposable Income neighbourhoods and in the city of Barcelona in 2005–07 (Pre-intervention A), 2008–10 (Pre-intervention B), 2011–13 (Post-intervention C) and 2014–16 (Post-intervention D). ^*^The intervention was carried out from October 2010 to July 2013 in the neighborhoods of Ciutat Meridiana, Torre Baró and Vallbona, and from September 2011 to October 2014 in El Bon Pastor and Baró de Viver. Common intervention years were 2011–13

Table 1 describes the intervention and comparison neighbourhoods by city Household Disposable Income (HDI), AFR, neighbourhood population and percentage of immigrant population. Figure 1 shows AFR in 2005–2007 (pre-intervention A), 2008–2010 (pre-intervention B), 2011–2013 (post-intervention C) and 2014–16 (post-intervention D).

At baseline, before the implementation of the SIRIAN program, the AFR was very high in the intervention group and high in the comparison group. With the community counselling implementation in 2011–13, intervention areas experienced large declines in 2011–16, while comparison neighbourhoods showed a small non-significant increase. Table 2 quantifies a decrease of − 12.30 (95% CI:-12.45 to − 12.21); *p* < 0.001) in the AFR of intervention neighbourhoods and the absence of significant changes in the comparison group (change in AFR: 1.91 (95% CI:-2.25 to 6.07; *p* = 0.368). An AFR reduction of − 10.97 per 1000 women 15–19 years (95% CI:-13.91 to − 8.03; *p* < 0.001) may be estimated as caused by the intervention. Relative differences were − 30.7 in the intervention group and 19.8 in the comparison group. The adjusted relative risks obtained with Poisson regression analysis were aRR = 0.54 (95% CI: 0.525–0.555) for the intervention group, aRR = 1.00 (95% CI: 0.980–1.020) for the comparison group and aRR = 1.852 (95% CI: 1.789–1.916) for the interaction, the DiD effect.

**Table 2:** Adolescent fertility rates (per 1000 women 15–19 years), relative difference, absolute change, adjusted change and difference in differences in the SIRIAN programme intervention and comparison neighbourhoods in the pre-intervention (2005–2010) and post-intervention periods (2011–16)

Neighbourhood group	AFR^a^ Pre 2005–10	AFR^a^ Post 2011–16	Relative difference^b^	Absolute change^c^ AFR^a^ (95%CI)	*p value*	Difference in differences^d^ AFR^a^ (95%CI)	*p value*	Adjusted change^c^ AFR^ae^ (95%CI)	*p value*	Difference in differences^d^ AFR^ae^ (95%CI)	*p value*
Intervention	27.90	19.34	−30.7	−8.56 (−13.22 to − 3.80)	< 0.001	−12.84 (− 20.67 to − 5.01)	< 0.01	− 12.30 (− 12.45 to − 12.21)	< 0.001	− 10.97 (− 13.91 to − 8.03)	< 0.001
Comparison	21.84	26.17	19.8	4.33 (−1.93 to 10.59)	0.175			1.91 (−2.25 to 6.07)	0.368		

^a^*AFR* Adolescent fertility rate (per 1000 women 15–19 years)

^b^Relative difference: (AFR Pre-AFR post/AFR Pre)*100

^c^β_**I**_ [Intervention]: Intervention and comparison groups

^d^β_T*I_ [Time*Intervention]: interaction between period and intervention

^e^Adjusted by annual neighbourhood’s immigrant population per group

The parallel test analysis showed not significant differences between the groups in the first pre-intervention segment (β_T*I_: 8.16 (95%CI): − 1.86 to 18.17; *p* = 0.110). In the analysis to assess whether the effect of the intervention decrease, no AFR differences appeared between the groups (β_T*I_:-0.04 (95%CI: − 10.84 to 10.76); *p* = 0.995) in the post periods C (2011–3) and D (2014–16).

In the sensitivity check, where we replicated the model with a comparison group including the rest of neighbourhoods in the lowest Disposable Household Income tercile of the city, this group of neighbourhoods showed apparently better trends parallelism before the intervention. Its pre-intervention rate (2005–10) was 13.57 per 1000 women 15–19 years old, and in post-intervention 11.94 (2011–16), yielding a relative difference of − 12.02%, without significant AFR differences (β_T_:-1.51(95%CI: − 3.56 to 0.54); *p* = 0.148). In the comparison of the intervention group with this 19 neighbourhoods comparison group, a significant interaction emerged (β_T*I_:-6.99 (95%CI:-1.86 to − 12.13); *p* = 0.008).

## Discussion

This study provides evidence for the possibility that the intervention reduced AFR. After the implementation of the SIRIAN program in neighbourhoods with low family income, there was a 30.7% reduction in their AFR, without significant changes in comparison neighbourhoods.

There are not many interventions with which to compare these results. A review of 21 evaluated interventions reported 9 statistically significant declines in teenage pregnancy rates (five cash transfer programs, one education curriculum, two life-skills curricula, and a provision of contraception intervention), while 7 reported increases in contraceptive use (three provision of contraception interventions, two life-skills curricula, a peer education program, and a mass media campaign), two reported decreases in sexual activity (a cash transfer program and an education and life-skills curriculum), and two cash transfer programs reported an increase in age of sexual debut [32]. None of them was based in brief individual contraceptive counselling in the community, although a Cochrane review concluded that the combination of educational and contraceptive-promoting interventions appeared to reduce unintended pregnancy among adolescents [13]. Another systematic review pointed out some potential in enhancing contraceptive use: it found 11 studies testing brief strategies for young people, a great heterogeneity across studies in participants’ ages and life situation, and, among the five studies with some effect, one provided moderate-quality evidence in increasing contraceptive use and four were older studies with low-quality evidence [14]. Our intervention was similar and the results were consistent with Safer Sex Intervention, an individual-level, clinic-based intervention implemented by an educator to reduce STIs and improve condom use among girls aged 13 to 20 years at high-risk for contracting an STI. It consisted of one initial session lasting 30–50 min and three booster sessions delivered at one, three, and 6 months. A randomized controlled trial comparing intervention groups from 2012 to 2015 described an overall significant increase of condom use at 9 months, as well as behavioural changes for Hispanic youth [33]. The main difference between this intervention and the SIRIAN program is the community dimension of the latter, which increases the access of newcomers and populations vulnerable to healthcare, without replacing it.

To put in context this intervention, the worldwide decrease in adolescent fertility in this century has to be taken into account [4]. Adolescent childbearing is on the decline in many countries, in particular where girls’ secondary school enrolment rates are going up [2, 34]. In the US, along with comprehensive strategies, teen pregnancy and birth rates declined dramatically over the past two decades, with a 64% reduction in the 2016 birth rates in comparison to the peak year 1991 [35], although US rates of teen childbearing still remain far higher than in most developed countries [4].

Some national initiatives such as the Teen Pregnancy Strategy in England have shown good results [5]. With an effort maintained during a decade in prevention work through service improvement, workforce training, promotion of enhanced choices of contraception, and mass-media campaigns, an observational evaluation described a 41% reduction in the under-18 conception rate from 1998 to 2014, with all local areas showing reductions in maternity and abortion rates [5]. In evaluations based in observational data it is difficult to disentangle the effects of policies from secular trends [36]: in the same periods declines were also seen in Scotland and Wales, where similar interventions to reduce teenage pregnancy rates were simultaneously organized, albeit branded differently [37].

Although our community program is not fully comparable with national strategies implemented in quite different contexts, some common points could be related to success: 1) the strategies were developed in small areas, 2) they were based in strong surveillance systems, 3) they had an approach involving both society and government, 4) they aimed to improve knowledge and access to the full range of contraception, 5) they were built on coordinated action between health care and public health services over a long period, and 6) they operated in the context of universal care provision by a National Health Service.

We would like to discuss whether the decrease of AFR in the most deprived areas of Barcelona could be attributed to broader social changes, such as secular trends, access to abortion, crisis and selective emigration or other interventions in the neighbourhoods. The possible effect of changes in immigration in small areas was taken into account in the analysis. About the secular trend, the fact that the comparison group did not show significant changes suggests that the estimates of the effect of SIRIAN can be attributed to the intervention. Another important explanation of the effect of the SIRIAN program on AFR could be changes in access to abortion in Spain, but during the intervention period there were no major changes in public access to emergency contraception or in abortion accessibility.

Another point to explore is the contribution of abortion in the effect of decreasing adolescent fertility. Although in the evaluation of the SIRIAN program we wanted to assess the differences in adolescent abortion rates between the intervention and comparison groups, we could not evaluate it because abortion data in Spain is not available by individual address, but only aggregated by postal codes, areas not related to the neighbourhoods. However, a visual examination of the abortion rate maps does not show apparent differential rates between the study groups (data not shown). Even though we would have liked to study eventual changes in population contraceptive use, the data were not available by neighbourhoods, and areas served by public family planning didn’t allow to separate the groups.

Important events, such as the impact of the economic recession, may have affected the effect of the intervention. As in other European countries, fertility declined in Spain during the economic recession in relation to the previous period, and the decline in fertility was generally deeper in regions with higher unemployment rates. The pathways through which the economic context postpones family formation among adolescents and youth include unemployment, the fall of job stability, uncertainty about the future, changes in housing markets, and also the prolonged enrolment in education and the delay in the formation of couples. This may have affected the city as a whole but there is no reason to infer differential results between the study groups, since both were in the same districts and with similar level of deprivation.

Regarding the comparability of the groups, as shown in Table 1 and Fig. 1, the AFR were very similar in both groups in 2008–10, but they seemed different in 2005–07. This initial difference in the AFR could be related to: a) the acute flow of immigration occurred in the first years of the decade: in 2001 there were 74,019 immigrants in Barcelona, in 2005 more than 230,000, and more than 280,000 in 2007; and b) to complex settlement flows that varied over time in this period. To take this into account, we adjusted the analysis by the percentage of the annual immigrant population in each neighbourhood. With respect to the effect of cultural diversity in the areas, in both study groups the composition of the neighbourhoods was mixed, being the first origin Latin American countries, followed by Pakistan and Morocco [9]. There were not other interventions addressing adolescent pregnancy in the study neighbourhoods (intervention and comparison) during 2011–16.

We would like to comment on the effects on the results of using the intervention period Jan 2011-Dec 2013 instead of the actual intervention period of Oct 2010-Oct 2014. As stated in methods, adolescent fertility was only available by complete years, and using the intervention period in 2011–13, the common years with intervention, allowed making comparisons with birth data. The eventual effects produced by the intervention beyond this period were included in the 2011–16 period, probably without further consequences. The eventual effects of the intervention from October 2010 to December 2010 in three neighbourhoods were included in the pre-intervention period (2005–10), and would have acted in favour of null hypothesis.

Relating to the methodology, DiD can be used in quasi-experimental designs and natural experiments, when two periods of data are available for the treatment and comparison groups. The DiD estimator measures the treatment effect by looking at the difference between the average outcome in the comparison and treatment groups, before and after treatment. A key assumption of DiD is the parallel trend assumption, which assumes that, in the absence of treatment, the average outcomes of the treatment group and the comparison group would follow parallel paths over time. In our study, in the Fig. 1 differences between the groups in the first pre-intervention segment appear to visually affect the parallel trend assumption, even though the differences were not significant.

The sensitivity check with a comparison group including the rest of neighbourhoods in the lowest Disposable Household Income tercile gave support to the results obtained in the main analysis, in which the comparison group included three similar neighbourhoods.

Selective migration may have been responsible for changes in AFR. The crisis may have stimulated a selective emigration of immigrant residents to their countries of origin in the intervention period, leading to an overestimation of the impact. To control this possibility, we adjusted the analyses by the percentage of immigrant population in both groups.

Reviewing the mechanisms of the intervention can also help inform the scope and credibility of the results. First, the program reached a 21.6% of the age group population in the intervention neighbourhoods. A 55.1% of the participants were girls and a 44.9% were immigrants. With a control randomized trial, the intervention demonstrated an increase of the use of contraceptives [16, 29]. Secondly, the magnitude of the effect of the program could be related to the health equity approach [38]. In addition, the development of the program benefited, paradoxically, from the lack of knowledge of the causes of these inequities, because international economic immigration and cultural diversity were, at that time, new issues in our country. For this reason, a large qualitative study was carried out to explore attitudes, knowledge and access to medical care for immigrants from different countries [10, 29]. This allowed identifying and act on specific psychosocial determinants of immigrants and native populations, and enhance access to reproductive care, linked to local providers. These cultural considerations may have increased the effects of the usual motivational interviews and counselling.

The intervention took into account the fact that the number of adolescent births in a relatively small number of families experiencing social and economic difficulties constitutes a high proportion of all AFR. Therefore, in each interview a modest incentive (a 10-trip travel card with a cost of €10) was provided to attract and retain the most disadvantaged participants in the follow-up [29]. This small behavioural incentive may have contributed to the impact on population indicators by focusing resources on those who need it most. In this way, the mechanisms to combat inequities could cover different socio-ecological levels. By affecting individual attitudes and behaviours, the intervention may have acted at the community level, changing the social norms of the vulnerable groups and empowering the communities to take control of a perceived need. Another possible contributing mechanism could be the fact that youth attitudes, knowledge and social norms could have been affected through interpersonal channels by older participants, women and men living in the same community.

The present study benefits from several strengths, including a quasi-experimental design, particularly useful when complex interventions occur in real-world settings [39, 40]; the choice of socioeconomic measures based on areas to identify comparable groups [41]; a well-established measure of the socioeconomic deprivation of neighbourhoods [19]; and the use of information systems of adequate quality available in Barcelona for decades. Moreover, the selection of disadvantaged urban neighbourhoods increased the generalizability of the results to similar contexts, cities and countries. Currently, the program has progressively expanded to other Barcelona neighbourhoods with unfavourable reproductive health indicators.

The main limitation of this study is its quasi-experimental design. As the disadvantaged areas were not randomized, the comparison groups may differ in some issues and affect the study internal validity. However, all the studied neighbourhoods were located within the same districts and the lowest tercile of deprivation of the city, without significant differences between the groups, which reinforces the assumption that they were areas with levels of similar deprivation. In addition, considering this is a natural experiment, a difference-in-difference method was used to reduce the risk of selection bias [42].

Another limitation reflects the difficulties of performing natural experiments. The fact that the adolescent fertility rates data were only available by units of complete years made impossible to match daily the intervention period, which began 3 months before 2011 in three intervention neighbourhoods and finished in 2014 in the other two intervention neighbourhoods. Even though, the exposure before 2011 operates against the intervention hypothesis.

To overcome the small number of adolescent births in neighbourhoods which were manifest in the numerator and in the population’s denominator (some had less than 2000 residents), which made year by year comparisons impossible, we added years to compare periods; as the intervention was implemented over 3 years, this increased statistical power and provided more stable estimates.

Finally, we would like to mention the feasibility, in terms of public health action, of this community intervention, which required a continuous but moderate effort over 3 years in training, implementing, monitoring and funding. Although the intervention appears to have caused a decrease in AFR, there are still high differences between some neighbourhoods, including those in the intervention group, and the rate of Barcelona, revealing inequalities to address. Attending the results of the intervention, the municipality replicated the SIRIAN program in 2017–18 in the 12 neighbourhoods with higher AFR rates in the city, including three of those who had already been intervened, and all those who were in the comparison group. Depending on the evolution of the rates and the costs, which have been estimated around €10,000 for 1 year of intervention per neighbourhood, as well as the opportunity costs in relation with other city health needs, the program may be replicated in the future, with adjustments to new population needs and contexts.

## Conclusion

This quasi-experimental study provides evidence that a community counselling intervention that increases access to contraception, knowledge and sexual health care in hard-to-reach segments of the population can contribute to substantially reduce AFR in urban areas of a large city of a Southern European country. Reducing AFR could become a feasible goal in cities with similar conditions.

## Data Availability

The datasets used and/or analysed during the current study are available from the corresponding author on reasonable request.

## Abbreviations

AFR: Adolescent fertility rate
HDI: Household Disposable Income
WHO: World Health Organisation

## References

[1] Romero L, Pazol K, Warner L (2016). Reduced Disparities in Birth Rates Among Teens Aged 15–19 Years — United States, 2006–2007 and 2013–2014. MMWR Morb Mortal Wkly Rep.

[2] Santelli JS, Song X, Garbers S, Sharma V, Viner RM (2017). Global trends in adolescent fertility, 1990–2012, in relation to national wealth, income inequalities, and educational expenditures. J Adolesc Health.

[3] Caffe S, Plesons M, Camacho AV (2017). Looking back and moving forward: can we accelerate progress on adolescent pregnancy in the Americas?. Reprod Health.

[4] United Nations. Adolescent fertility trends. Population facts. //data.worldbank.org/indicator/SP.ADO.TFRT. Published 2013. Accessed 30 May 2018.

[5] Wellings K, Palmer MJ, Geary RS (2016). Changes in conceptions in women younger than 18 years and the circumstances of young mothers in England in 2000–12: an observational study. Lancet.

[6] Alkema L, Kantorova V, Menozzi C, Biddlecom A (2013). National, regional, and global rates and trends in contraceptive prevalence and unmet need for family planning between 1990 and 2015: a systematic and comprehensive analysis. Lancet.

[7] Cleland J, Conde-Agudelo A, Peterson H, Ross J, Tsui A (2012). Contraception and health. Lancet.

[8] Díez E, Vadillo V, Cabanas M EL. Estudi qualitatiu dels determinants de la salut reproductiva en dones immigrades. In: La Salut a Barcelona 2004. Barcelona: Agència de Salut Pública de Barcelona; 2005. p.54-61. https://www.aspb.cat/wp-content/uploads/2016/03/InformeSalut-2004.pdf.

[9] Ajuntament de Barcelona. Població Estrangera a Barcelona: Informació Sociodemogràfica. Barcelona: Agència de Salut Pública de Barcelona, 2013. http://www.bcn.cat/novaciutadania/pdf/ca/estudis/pob_estrangera_2013.pdf.

[10] Agència de Salut Pública. La Salut a Barcelona 2005. Barcelona: Agència de Salut Pública de Barcelona, 2006. http://www.aspb.cat/wpcontent/uploads/2016/03/informe-salut-2005.pdf.

[11] Agència de Salut Pública de Barcelona. La Salut a Barcelona 2008. Barcelona: Agència de Salut Pública de Barcelona, 2009. https://www.aspb.cat/wpcontent/uploads/2016/03/Salut_bcn_2008.pdf.

[12] Benaque A, Borrell C, Nebot M, Díez E. Teenage maternity in the districts and neighbourhoods of Barcelona: its association with low social and economic level, and the prevalence of low birth weight. Atención Primaria. 1997;19:449-54. https://www.elsevier.es/es-revista-atencion-primaria-27-articulo-maternidad-adolescentes-los-distritos-barrios-14552. 9264678

[13] Gilliam ML (2010). Interventions for preventing unintended pregnancies among adolescents. Obstet Gynecol.

[14] Lopez LM, Grey TW, Tolley EE, Chen M (2016). Brief educational strategies for improving contraception use in young people. Cochrane Database Syst Rev.

[15] Díez E, López MJ, Marí-Dell’olmo M (2018). Effects of a counselling intervention to improve contraception in deprived neighbourhoods: a randomized controlled trial. Eur J Pub Health.

[16] Nebot L, Diez E, Martin S (2016). Efectos de una intervencion de consejo anticonceptivo en adolescentes de barrios desfavorecidos con alta proporcion de inmigrantes. Gac Sanit.

[17] Cook TD, Campbell DT (1979). Quasi Experimentaion: Desgin & Analysis Issues for Field Settings.

[18] Barcelona Economia (2012). Distribució territorial de la renda familiar disponible per càpita a Barcelona (2011).

[19] OECD. OECD Factbook 2015-2016. Economic, Environmental and Social Statistics. Paris: OECD Publishing; 2016. p. 50. 10.1787/factbook-2015-en.

[20] Perez G, Borrell C. Manual d’Elaboració i Explotació Del Sistema d’Informació de Salut Reproductiva de Barcelona. Barcelona: Agència de Salut Pública de Barcelona; 2011.

[21] WHO. Brief Sexuality-Related Communication: Recommendations for a Public Health Approach. Geneva: WHO; 2015. http://www.who.int/reproductivehealth/publications/sexual_health/sexuality-related-communication/en/. 26290928

[22] Lopez LM, Grey TW, Chen M, Tolley EE, Stockton LL (2016). Theory-based interventions for contraception. Cochrane Database Syst Rev.

[23] Hicking-Woodison L (2017). Planning health promotion programs: an intervention mapping approach.

[24] Agència de Salut Pública de Barcelona. Barcelona Salut Als Barris. Diagnòstic Torre Baró, Ciutat Meridiana i Vallbona. Barcelona: Agència de Salut Pública de Barcelona; 2010. https://www.aspb.cat/wp-content/uploads/2016/07/Diagnostic_salut_ZNord_2009.pdf.

[25] Agència de Salut Pública de Barcelona. Barcelona Salut Als Barris. Diagnòstic Bon Pastor I Baró de Viver. Barcelona: Agència de Salut Pública de Barcelona; 2011. https://www.aspb.cat/documents/salut-als-barris-diagnostic-salut-bon-pastor-baro-viver/.

[26] World Health Organization (2007). Introducing WHO’s Sexual and Reproductive Health Guidelines and Tools into National Programme: Principles and Processes of Adaptation and Implementation.

[27] Wilson A, Nirantharakumar K, Truchanowicz EG, Surenthirakumaran R, MacArthur C, Coomarasamy A (2015). Motivational interviews to improve contraceptive use in populations at high risk of unintended pregnancy: a systematic review and meta-analysis. Eur J Obstet Gynecol Reprod Biol.

[28] Lopez LM, Otterness C, Chen M, Steiner M, Gallo MF (2013). Behavioural interventions for improving condom use for dual protection. Lopez LM, ed. Cochrane Database Syst Rev.

[29] Diez E, López MJ, Marı-Dell’Olmo M (2018). Effects of a counselling intervention to improve contraception in deprived neighbourhoods: a randomized controlled trial. Eur J Public Heal.

[30] Díez E, Oliva C, Cortés J, Cobo E, Gómez S, Vadillo V, Cabanas M, Barcons N, Perez G, Foz M, Estruga Ll, Almirall R, Vela E, Martínez C. Artazcoz L, Pidelaserra F, Lopez S, Cabús E. Promoció de la contracepció en dones immigrades i autòctones de Barcelona: anàlisi intermèdia del programa SIRIAN. Barcelona Soc. 2009;16:89-102. https://ajuntament.barcelona.cat/dretssocials/ca/barcelona-societat-num-16.

[31] Zhou H, Taber C, Arcona S, Li Y (2016). Difference-in-differences method in comparative effectiveness research: utility with unbalanced groups. Appl Health Econ Health Policy.

[32] Kalamar AM, Lee-Rife S, Hindin MJ (2016). Interventions to prevent child marriage among young people in low- and middle-income countries: a systematic review of the published and gray literature. J Adolesc Health.

[33] Kelsey M, Walker JT, Layzer J, Price C, Juras R (2016). Replicating the safer sex intervention: 9-month impact findings of a randomized controlled trial. Am J Public Health.

[34] Entre Nous (2014). Adolescence : building solid foundations for lifelong flourishing. Entre Nous.

[35] Hamilton BE, Martin JA, Osterman MJKS, Driscoll AK, Rossen LM (2018). Births: provisional data for 2016. Vital Stat Rapid Release.

[36] Chen Y-F, Hemming K, Stevens AJ, Lilford RJ (2016). Secular trends and evaluation of complex interventions: the rising tide phenomenon. BMJ Qual Saf.

[37] Craig P, Dundas R, Leyland A, Popham F (2016). How successful was the English teenage pregnancy strategy?. Lancet.

[38] Whitehead M (2007). A typology of actions to tackle social inequalities in health. J Epidemiol Community Health.

[39] Kontopantelis E, Doran T, Springate DA, Buchan I, Reeves D (2015). Regression based quasi-experimental approach when randomisation is not an option: Interrupted time series analysis. BMJ.

[40] Craig Peter, Cooper Cyrus, Gunnell David, Haw Sally, Lawson Kenny, Macintyre Sally, Ogilvie David, Petticrew Mark, Reeves Barney, Sutton Matt, Thompson Simon (2012). Using natural experiments to evaluate population health interventions: new Medical Research Council guidance. Journal of Epidemiology and Community Health.

[41] Ontario Agency for Health Protection and Promotion (Public Health Ontario) (2013). Summary Measures of Socioeconomic Inequalities in Health.

[42] White H, Sabarwal S. Quasi-Experimental Design and Methods. Florence; 2014. https://www.unicef-irc.org/publications/pdf/brief_8_quasi-experimentaldesign_eng.pdf

